# Implications of Inter-Individual Differences in Clopidogrel Metabolism, with Focus on Pharmacogenetics

**DOI:** 10.3390/ph3040782

**Published:** 2010-03-24

**Authors:** Marja-Liisa Dahl, Arzu Gunes

**Affiliations:** 1 Department of Medical Sciences, Clinical Pharmacology, Uppsala University, Uppsala, Sweden; 2 Department of Clinical Pharmacology, Karolinska University Hospital, Huddinge C1:68, SE-141 86, Stockholm, Sweden

**Keywords:** clopidogrel, CYP2C19, antiplatelet response, pharmacogenetics

## Abstract

Increasing evidence for the role of pharmacogenetics in treatment resistance to the antiplatelet agent clopidogrel has been gained during the last years. Apart from *CYP2C19* genetic polymorphisms, nongenetic factors, particularly drug-drug interactions, age and other clinical characteristics influence the interindividual variability in clopidogrel response to varying degrees. The present article reviews the so far accumulated evidence on the role of pharmacogenetic traits influencing CYP-activity as determinants of the antiplatelet response to clopidogrel, and its clinical implications. The genetic variation in CYP2C19 activity seems to influence short- and long-term antithrombotic effects of clopidogrel to a substantial extent. Prediction models for clopidogrel non-responsiveness that include *CYP2C19* genotyping together with relevant non-genetic risk factors are needed to be verified for their potential benefit in individualization of antithrombotic therapy.

## 1. Introduction

The antiplatelet agent clopidogrel is used in combination with aspirin in the treatment of patients with acute coronary syndrome and those undergoing percutaneus coronary intervention (PCI) to prevent ischemic events and stent thrombosis. However, there is large interindividual variability in the response to clopidogrel and 15–40%, depending on the criteria used, are considered as non-responders, or clopidogrel-resistant, with high residual platelet aggregation [1]. There is today vast evidence indicating that high platelet reactivity despite clopidogrel treatment is a risk factor for cardiovascular events and stent thrombosis [2,3].

**Figure 1:**
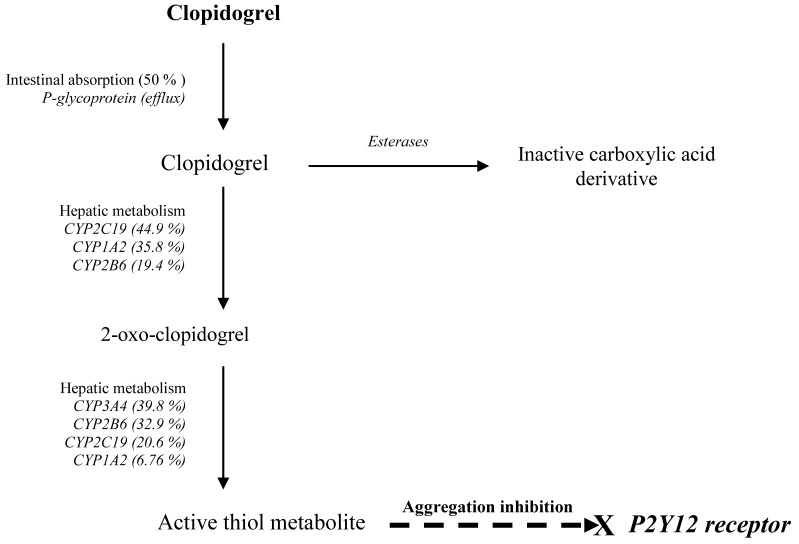
Pharmacokinetic and pharmacodynamic pathways involved in clopidogrel response.

Clopidogrel is an inactive pro-drug that requires oxidation to its active thiol metabolite. The active metabolite inhibits platelet aggregation irreversibly by blocking platelet P2Y12 receptors, resulting in reduced adenosine 5´-diphosphate (ADP)-mediated activation of the glycoprotein GPIIb/IIIa complex [4]. About 85% of clopidogrel is hydrolysed via esterases to an inactive carboxylic acid derivative and only about 15% undergoes hepatic cytochrome P450 (CYP)-catalysed metabolism to a 2-oxo-clopidogrel intermediate that is subsequently oxidized to the active metabolite, a thiol derivative of clopidogrel [4]. *In vitro*, the first step is catalyzed by several enzymes including CYP2C19, CYP1A2 and CYP2B6 and the second by CYP3A4/5, CYP2B6, CYP2C19 and CYP2C9 [5] (Figure 1). *In vivo*, CYP3A4, CYP2C19 and CYP1A2 are considered the main enzymes involved. All these enzymes show large inter-, and sometimes intraindividual variability in activity, due partly to genetic polymorphisms, partly to non-genetic factors such as inhibition or induction by other drugs, herbal medicines and other environmental factors. These factors can thus potentially influence the exposure to the active clopidogrel metabolite and, subsequently, its antiplatelet effect. In the present article we review the evidence on the role of pharmacogenetic traits influencing CYP-activity as a determinant of the antiplatelet response to clopidogrel and its clinical implications. 

## 2. CYP2C19 and Clopidogrel Pharmacokinetics and Pharmacodynamics

CYP2C19 is one of the major enzymes involved in both the formation of the intermediate metabolite, 2-oxo-clopidogrel, and its further metabolism to the active thiol metabolite (Figure 1). Functional polymorphisms in the *CYP2C19* gene result in highly variable enzyme activity. Poor metabolisers (PM), encompassing 2–4% of Caucasian and 14–20% of Asian populations, completely lack CYP2C19 activity [6]. The CYP2C19*2 allele accounts for the majority of PM, whereas in Asia, CYP2C19*3 also contributes to this phenotype. Other more rare alleles causing deficient metabolism are CYP2C19*4, *5, *6, *7 and *8 [7]. The CYP2C19*17, on the other hand, results in increased enzyme activity [8] and is found with a frequency of 18–27% in European populations [9,10,11] but less frequently (1.3%) in Asians [12]. 

Significant associations between *CYP2C19* genotype and differences in pharmacokinetics of clopidogrel and its active metabolite, and the antiplatelet response to clopidogrel have been identified in a number of studies in healthy volunteers of both Caucasian and Asian ethnicity [13,14,15,16] (Table 1). A 30–50% decrease in plasma exposure (AUC) and maximum plasma concentration (Cmax) of the active metabolite in carriers of the defective CYP2C19*2 allele compared to those with the CYP2C19*1*1 genotype were associated with lower inhibition of platelet aggregation following 300 and 600 mg loading doses as well as after 75 mg maintenance doses [13,14,15,16]. Two further studies [17,18] in Caucasian subjects, lacking pharmacokinetic endpoints, showed similar results, with a significantly decreased platelet responsiveness to clopidogrel in subjects carrying the CYP2C19*2 allele as compared to those with CYP2C19*1*1 genotype. The relative difference in antiplatelet response between genotype groups varies across studies depending on the method used to evaluate response, but is typically greater than 30%. The CYP2C19*2 carrier status reportedly explains around 10% of the variability in clopidogrel response [18]. A gene-dose relationship has been shown in studies in Asian populations, where higher numbers of subjects homozygous for *CYP2C19* alleles coding for defective enzyme (e.g., PM) are found [6].

**Table 1:** The influence of *CYP2C19* genotype (heterozygous or homozygous carriers of defect alleles compared to *1*1 genotype) on the pharmacokinetics (PK) and pharmacodynamics (PD) of clopidogrel.

Study population	Treatment dose (mg)	Impact of *CYP2C19* genotype on PK and PD			Reference
		Parameter	Heterozygous ^a^	Homozygous ^b^	
HV Dutch mostly Caucasian (n =74)	300	*Active metabolite*			[13]
		AUC_0–24_	1.8–fold ↓	2.8– fold ↓	
		Cmax	1.7–fold ↓	2.1–fold ↓	
		*PA inhibition*			
		(induced by 20 µM ADP)	1.9–fold ↓	10.3–fold ↓	
HV Mixed (n =162)	300	*Active metabolite*			[14]
		AUC	1.4–fold ↓	2.2–fold ↓	
		*PA inhibition*	1.1–fold ↓	1.2–fold ↓	
		(induced by 20 µM ADP)			
	600	AUC	1.4–fold ↓	NA	
		*PA inhibition*	1.1–fold↓	NA	
		(induced by 20 µM ADP)			
	75	AUC	1.1–fold↓	1.8–fold↓	
		*PA inhibition*	1.4–fold↓	1.4–fold↓	
		(induced by 20 µM ADP)			
HV Japanese (n =47)	300	*Active metabolite*			[15]
		AUC_0-8_	1.4–fold ↓	1.8–fold ↓	
		Cmax	1.5–fold ↓	1.6–fold ↓	
		*PRI*			
		(4 h after dosing)	1.2– fold ↑	1.4–fold ↑	
HV Korean (n =24)	300	*Clopidogrel*			[16]
		AUC_0–24_	1.8–fold ↑	2.9–fold ↑	
		Cmax	1.8– fold↑	4.7–fold ↑	
		*PA inhibition*			
		(induced by 5 µM ADP)	1.1–fold ↓	2.2–fold ↓	

[13] ***^a^*** CYP2C19*1*2 **^b^**CYP2C19*2*2

[16] ***^a^*** CYP2C19*1/*2, *1/*3 **^b^** CYP2C19 *2/*2, *2/*3

[15] ***^  a^*** CYP2C19*1/*2, *1/*3 **^b^** CYP2C19*2/*2, *2/*3

[14] **^a^** CYP2C19*1/*2, 1/*3, *1/*4, *1/*8 **^b^** CYP2C19*2/*2, *2/*3, *2/*4, *2/*5, *2/*8

HV; healthy volunteers, PA; platelet aggregation, PRI; platelet reactivity index

The relationship between *CYP2C19* genotype and platelet response to clopidogrel has been confirmed in a number of studies in patients (both Caucasian and Asian) with acute coronary syndrome or undergoing PCI [19,20,21,22,23,24,25]. In only one of them, the plasma levels of the active metabolite of clopidogrel were analysed [25]. Various methods were used to assess platelet function and a few focused on clopidogrel non-response, also defined in various ways. In the latter studies, defective *CYP2C19* alleles (*2 or *3) were more prevalent in the clopidogrel resistant group than in the responsive group [19,23]. Even after adjustment for other factors influencing platelet responsiveness such as age, gender, hypertension, diabetes mellitus, and smoking status, CYP2C19*2 [19] and *3 [23] remained significant and independent risk factors for impaired antiplatelet effect of clopidogrel.

The CYP2C19*17 variant is associated with increased enzyme activity [8]. Accordingly, an enhanced antiplatelet effect of clopidogrel could be expected in subjects carrying this variant allele. Carriers of CYP2C19*17 were shown to have a trend towards the highest plasma exposure to the active metabolite after 300 and 600 mg loading and 75 mg maintenance doses, together with the highest reduction of maximal platelet aggregation 24 hours after the administration of clopidogrel [14]. In another study, no difference in residual platelet aggregation was detected between either hetero- or homozygous carriers of CYP2C19*17 as compared to CYP2C19*1*1 carriers after a 600 mg loading dose of clopidogrel [19]. In a third study, in patients with non-ST elevation acute coronary syndrome, a lower rate of non-responders was found among CYP2C19*17 carriers as compared to subjects not carrying this allele [26]. However, no impact of the CYP2C19*17 allele on ADP-induced platelet aggregation was observed. In one further study, CYP2C19*17 carriers unexpectedly had decreased platelet inhibition (measured by Verifynow P2Y12 analyser), similar to that in carriers of defect alleles, compared to CYP2C19*1*1 carriers 2 hours after a 600 mg dose of clopidogrel [22]. Unfortunately, no plasma concentration data were available in the latter studies. However, taken together, the role of CYP2C19*17 in clopidogrel response appears marginal.

Recently, a genome wide association study was performed to identify markers of clopidogrel response, in 429 healthy Amish individuals administered 300 mg oral loading dose of clopidogrel followed by 75 mg daily for 6 days [27]. Interestingly, the strongest association with diminished clopidogrel response was found for a polymorphism (rs12777823) that is in linkage disequilibrium with CYP2C19*2 (r^2^ = 0.87). Age, BMI, triglyceride and high-density lipoprotein cholesterol levels were also associated with clopidogrel response. The variation in ADP-induced platelet aggregation explained by a combination of these clinical variables was less than 10%, while CYP2C19*2 accounted for** 12% of the variation. 

## 3. CYP2C19 Genotype and Long-Term Outcome of Clopidogrel Treatment 

A number of recent studies [14,27,28,29,30,31,32] in patients with acute coronary syndrome or PCI have demonstrated that the *CYP2C19* genotype-related antiplatelet effects of clopidogrel translate into clinically relevant differences in major cardiovascular events (stent thrombosis, death, myocardial infarction, and stroke) in patients followed up to one year. 

Trenk *et al.* [28] found in a study in 797 patients with PCI that carriers of CYP2C19*2 were more prone to have high residual platelet reactivity on clopidogrel than patients homozygous for the CYP2C19*1 allele. High residual platelet reactivity was in turn associated with a 3-fold increase in 1-year incidence of death and myocardial infarction. In another study in 227 patients undergoing PCI, carriers of CYP2C19*2 were more likely to have a cardiovascular ischemic event or death during 1 year follow-up (20.9% *vs*. 10.0% in CYP2C19*1*1, hazard ratio 2.42) [27]. In a large cohort of 1477 patients with acute coronary syndromes treated with clopidogrel, the *CYP2C19* defective allele carriers (*2, *3, *4, *5 or *8) had a 53% relative increase in the risk of death from cardiovascular causes, myocardial infarction or stroke, and a 3-fold increase in the risk of stent thrombosis, compared to non-carriers [14]. Similar results, with hazard ratios typically around 2–3 for stent thrombosis or death in CYP2C19*2 carriers, were reported in four further studies [27,30,31,32], while Simon *et al.* [29] only found a significantly higher event rate (adjusted hazard ratio 1.98) in patients carrying two defective alleles of *CYP2C19* (*2, *3, *4 or *5). 

The possible impact of *CYP2C19* genotype on bleeding complications during clopidogrel treatment has not been evaluated in previous studies with the exception of the study by Mega *et al.* [14] in which no association was found. 

## 4. Other CYP and ABCB1 Genotypes in Relation to Clopidogrel Response

The potential impact of polymorphisms in genes coding for other CYPs involved in clopidogrel metabolism (CYP1A2, 3A4/5, 2B6, 2C9) as well as for P-glycoprotein, an active transport protein, potentially influencing clopidogrel absorption, have been assessed in a number of studies in healthy volunteers and patients. CYP3A4 is expressed in both liver and gut and shows large inter- but also intraindividual variation, being subject to induction and inhibition by a number of drugs and other exogenous compounds. Even though a number of polymorphisms in the *CYP3A4* gene have been identified, no clear relationship between the genotype and CYP3A4 activity has been demonstrated. Only a minority of individuals express CYP3A5 due to the low frequency of the functional CYP3A5*1 allele [33,34]. 

So far, only one *CYP3A4* polymorphism, the IVS10+12G>A, has been reported to be associated with reduced glycoprotein IIb/IIIa activation but not with corresponding changes in platelet aggregation in patients treated with clopidogrel [2]. However, three later studies failed to repeat this association [18,20,24]. The impact of *CYP3A5* polymorphisms on platelet aggregation in response to clopidogrel has been studied in healthy volunteers and patients, with no difference in the antiplatelet activity being found between carriers of CYP3A5*1 (required for expression of the enzyme) and those with CYP3A5*3*3 genotype (with no CYP3A5 expression) [13,19,22]. However, in a Korean study in 348 patients with coronary angioplasty it was found that atherothrombotic events occurred more frequently within 6 months after stent implantation among patients with the non-expressor genotype (CYP3A5*3*3) than among those with the expressor genotype (CYP3A5*1*1 or *1*3) [35]. CYP3A5*3*3 genotype, together with the number of co-administered CYP3A substrates or inhibitors and total cholesterol levels of 5.18 mmol/L or higher, were found as independent predictors of atherothrombotic events. Close linkage disequilibrium has been shown between *CYP3A4* and *CYP3A5* polymorphisms, and hence the phenotypic associations of *CYP3A4* polymorphisms are hypothesised to be partly attributable to the contribution of CYP3A5 expression [36].

*CYP2C9* polymorphisms leading to decreased enzyme activity have been evaluated in several studies in relation to clopidogrel active metabolite exposure, inhibition of platelet aggregation and response [13,14,22,25]. Brandt *et al.* [13] reported decreased active metabolite exposure, lower inhibition of platelet aggregation and poor responder status in healthy volunteers with *CYP2C9* decreased function alleles (*2, *3, or *11) after the administration of 300 mg clopidogrel [13]. However, in three other studies, no impact of the *CYP2C9* genotype on the pharmacokinetic and pharmacodynamic responses to clopidogrel was found [14,22,25]. 

Other candidate genes assessed with respect to their potential influence on clopidogrel response are *CYP2B6* and *CYP1A2*. A tendency towards lower plasma exposure to the active metabolite of clopidogrel together with lower inhibition of platelet aggregation was found in healthy subjects carrying reduced function *CYP2B6* alleles (*6, *9 or *13) while there was no association between the *CYP2B6* genotype and clinical outcome in patients [14]. The lack of association was shown in another study [25]. So far, no impact of *CYP1A2* genotype on clopidogrel response has been shown [13,14,23]. 

The adenosine triphosphate–binding cassette efflux transporter ABCB1, also known as P-glycoprotein, is expressed in various tissues including the gut and potentially influences the bioavailability of many drugs. Several polymorphisms in the *ABCB1* gene have been identified, one of the most commonly studied being 3435 C > T which, despite being non-coding, has been linked to changes in P-glycoprotein expression and pharmacokinetics of a number of drug substrates. *In vitro* data indicate that clopidogrel is a substrate of P-glycoprotein [37]. In the same study, patients with the *ABCB1* 3435T/T genotype had lower Cmax and AUC values of both clopidogrel and the active metabolite compared to 3435C/T and 3435C/C carriers following 300 and 600 mg but not 900 mg loading doses. Thus, an enhanced intestinal efflux was implied in 3435T/T carriers. In concordance with this, Simon *et al.* [29] reported that patients with *ABCB1* 3435 T/T genotype had a higher rate of cardiovascular events at 1 year than those with *ABCB1* C/C genotype (adjusted hazard ratio 1.72 with 95% CI 1.29–2.47) [29]. However, as *ABCB1* genotype has not been found to be an independent predictor of the outcome, the result should be considered exploratory, requiring replication. In other studies where the *ABCB1* genotype has been analysed, no differences in platelet aggregation or outcome related to the genotype have been found [23,27].

## 5. Drug Interactions

Apart from genetic polymorphisms, interindividual differences in clopidogrel metabolism and response could be influenced by drug interactions involving inhibition or induction of the enzymes involved, most notably CYP3A and CYP2C19. In this context, the potential inhibitory effects of lipophilic statins on CYP3A4 and of proton pump inhibitors on CYP2C19 have gained wide attention. In 2003, Lau *et al*. [38] reported in a small study that atorvastatin, a CYP3A4 substrate, but not pravastatin which is metabolized by conjugation, reduced the inhibitory effect of clopidogrel on platelet aggregation in a dose-dependent manner. While a few reports have confirmed this finding, several others have not [39,40]. Whether some statins interfere with clopidogrel metabolism and response in a clinically relevant way remains controversial. Ketoconazole and itraconazole, both potent inhibitors of CYP3A4 decrease the formation of clopidogrel active metabolite together with reduced inhibition of platelet aggregation in healthy volunteers [40]. With respect to CYP2C19, omeprazole has been shown to decrease the inhibitory effect of clopidogrel on platelet aggregation in patients with coronary artery stenting [41]. In a population-based nested case control study concomitant use of proton pump inhibitors was associated with a 40% relative increase in the risk of reinfarction [42]. An exception was pantoprazole, a less potent CYP2C19 inhibitor than omeprazole. As with statins, the interaction potential of individual drugs within a group may differ and should be studied on a drug-specific basis. Another large retrospective cohort study [43] also found an increased risk of adverse outcomes among patients with concomitant use of clopidogrel and proton pump inhibitors (mainly omeprazole) after hospital discharge for acute coronary syndrome, compared to patients with clopidogrel but without proton pump inhibitors. However, prospective studies are needed to confirm these findings and to assess the causality of the associations found. 

## 6. Clinical Implications

In summary, genetic variation in* CYP2C19* gene and especially the deficient CYP2C19*2 variant has consistently been shown to result in significant differences in the exposure to the active metabolite of clopidogrel (Table 1), with associated changes in platelet aggregation and higher cardiovascular event rates in patients with the CYP2C19 PM status. Typically, hazard ratios in the range of 2–3 for these events in subjects carrying defective *CYP2C19* alleles have been reported, with follow-up periods up to 1 year. This was also acknowledged in 2009 by the US FDA, issuing a recommendation on an update to the label for Plavix. 

Genotyping for loss-of-function *CYP2C19* variants could thus identify individuals at risk for inadequate response during normal clopidogrel dosing. However, drug response is a multifactorial trait, and *CYP2C19* genotype alone only explains 10–12% of the variability in platelet response. Interestingly, in the study by Geisler *et al.* [19], a nomogram based on non-genetic risk factors (PREDICT-score) and the *CYP2C19* genotype was developed, to estimate the risk of individual patients for high residual platelet aggregation in response to a 600 mg loading dose of clopidogrel. This model has, to our knowledge, not yet been tested in a clinical setting. 

The clinical outcome studies published so far have mainly included patients of Caucasian origin. As the frequency of defective *CYP2C19* alleles, and that of subjects with complete lack of enzyme activity (PM phenotype), is much higher among Asians, the risk for clopidogrel non-response is potentially more important in those populations. However, other factors, both genetic and non-genetic, of importance to clopidogrel response may also vary between ethnic groups. Thus, large studies in ethnically diverse populations are warranted. 

Apart from merely identifying risk of non-response, pharmacogenetic testing could be used to identify patients in whom alternative antiplatelet treatments or other dosing strategies could be applied. The standard dosing schedule recommended includes a 300 mg loading dose followed by 75 mg maintenance dose. However, a 600 mg loading dose enhances the antiplatelet efficacy compared to 300 mg [44] and is commonly used. Higher doses of 900 mg, however, have been reported not to result in any further increase in plasma levels of clopidogrel, its active metabolite or the antiplatelet effect of the drug [44]. This ceiling effect has been attributed to saturable clopidogrel absorption at high doses. Interestingly, we found that CYP2C19*2 and *4 carriers, but not those with CYP2C19*1*1 genotype, showed significantly increased platelet inhibition response with higher loading doses (1200 mg *versus* 600 mg) and with higher maintenance doses (150 mg *versus* 75 mg) of clopidogrel [22]. This suggests that higher doses might improve clopidogrel response in subjects with impaired CYP2C19 activity. In a case series of 7 patients with stent thrombosis on 75 mg maintenance dose of clopidogrel, 6 of whom were carriers of CYP2C19*2 [45], incremental increases in maintenance dose up to 225 mg did result in slightly improved platelet inhibition in a dose-dependent manner. However, only 2 patients could be classified as responders at a dose of 300 mg which also resulted in side effects precluding continuation of treatment. Thus, apart from being time-consuming, dose titration was only marginally effective. Switch to prasugrel, an antiplatelet agent not metabolised by CYP2C19, on the other hand, resulted in a significant improvement of platelet inhibition in these patients. It is also to be pointed out that there is a large variation in platelet response within each genotype group, only a proportion of CYP2C19*2 carriers having the non-response phenotype, and a significant overlap between genotype groups. Not all patients with the “risk genotype” would thus necessarily require or benefit from alternative treatment strategies. Another aspect to be considered is the clinical relevance of clopidogrel “non-response” or “resistance” still under debate, partly owing to variations in the methodology and criteria used to define the phenotype. In particular, there is still lack of consensus regarding the definition of cut-off levels for tests of platelet function, time of sampling, as well as variations in the performance of tests between laboratories [46].

In summary, there is by now substantial documentation supporting the role of genetic variation in CYP2C19 activity as a determinant of the antiplatelet effects of clopidogrel, as well as clinical outcome in terms of thrombotic events. Genotyping for *CYP2C19* together with assessment of non-genetic risk factors could thus potentially improve the prediction of individuals with risk of clopidogrel non-responsiveness. Antiplatelet treatment tailored on the basis of this pharmacogenetic knowledge, but also taking non-genetic risk factors into account thus offers a promising area for future prospective studies of individualized antithrombotic therapy. 
